# Supplementary material for: Bet-hedging strategies in expanding populations
Source: PLoS Comput Biol. 2019 Apr 18;15(4):e1006529. doi: 10.1371/journal.pcbi.1006529 (PMC6490941; doi:10.1371/journal.pcbi.1006529)
Supplement: S1 Appendix — (PDF) [file pcbi.1006529.s001.pdf]

Supporting information: Bet-hedging strategies in expanding populations

Paula Villa Martín
Biological Complexity Unit, Okinawa Institute of Science and Technology Graduate University, Onna, Okinawa 904-0495, Japan

Miguel A. Muñoz
Departamento de Electromagnetismo y Física de la Materia and Instituto Carlos I de Física Teórica y Computacional, Facultad de Ciencias, Universidad de Granada, 18071 Granada, Spain

Simone Pigolotti*
Biological Complexity Unit, Okinawa Institute of Science and Technology Graduate University, Onna, Okinawa 904-0495, Japan.
(Dated: April 2, 2019)

NUMERICAL INTEGRATION OF THE STOCHASTIC FISHER EQUATION

In this section we describe in details the methods for the integration of the wave equations of the two-phenotype model used for simulations in the Main Text.

Fisher wave

We consider the Fisher equation

$$\dot{f}(x,t) = D\nabla^2 f(x,t) + \sigma(x,t) f(x,t)(1 - f(x,t)), \tag{1}$$

where $f(x,t)$ is the population density at space x and time t, and $\sigma(x,t)$ is the local growth rate.

We employ a finite-difference fourth-order Runge-Kutta method. The systems is initialized by fixing $f(x_i, 0) = 1$ for $i \in (0, 50)$ and $f(x,t) = 0$ for $i > 50$. The spatial mesh dx is fixed by means of an adaptive routine. We intialize the routine with an initial guess $dx = 0.14$. Then

1. We let the system evolve until the front reaches a stationary state.
2. We compute the smallest values of x for which $f(x,t) > \theta$ for $\theta = 3/4$ and $\theta = 1/4$. We denote these two values as $x_{3/4}$ and $x_{1/4}$ respectively.
3. We measure the precisions $\Delta f_{3/4} = f(x_{3/4} - dx) - f(x_{3/4})$, $\Delta f_{1/4} = f(x_{1/4} - dx) - f(x_{1/4})$.
4. If $\Delta f_{3/4} > 0.01$ and $\Delta f_{1/4} > 0.01$, then dx is accepted as a valid increment.
5. Otherwise, the system is reset to the initial condition and the routine is again run for $dx = d\tilde{x} - 0.01$; being $d\tilde{x}$ the previous spatial mesh.

Once dx is determined, dt is fixed following the Courant-Friedrichs-Lewy condition for an explicit integration method [1]:

$$\frac{v_{\max} dt}{dx} \leq 1 \tag{2}$$

* simone.pigolotti@oist.jp

being $v_{\max}$ the estimated maximum velocity of the wave. We fix $v_{\max} = 100$, which is sufficiently large compared to velocities in our simulations.

Temporal environmental switch is numerically implemented with a simple first-order algorithm. At the beginning of each time step, the state of environment is switched with probability $k\,dt$. We verified that this quantity is always sufficiently small, so that the first-order algorithm yields reliable results. A similar algorithm is implemented for spatial environmental variations to sequentially assign an environmental state to each lattice site.

Stochastic Fisher wave

We consider the stochastic Fisher equation [2]

$$\dot{f}(x,t) = D\nabla^2 f + \sigma(t) f(1-f) + \sqrt{\frac{2}{N} f(1-f)}\,\xi(x,t) \tag{3}$$

where $\xi(x,t)$ a Gaussian white noise satisfying $\langle \xi(x,t)\rangle = 0$ and $\langle \xi(x,t)\xi(x',t')\rangle = \delta(x-x')\delta(t-t')$. For later convenience, we define the noise amplitude $\gamma(t) \equiv \sqrt{\frac{2}{N} f(t)(1-f(t))}$.

Numerical integration in the presence of noise is subtle. In particular, one has to figure out how to deal with the unphysical values $f(x,t) < 0$ and $f(x,t) > 1$ that can result due to finite numerical precision. Depending on parameter range, the naive replacement $f(x,t) = 0$ or $f(x,t) = 1$ when $f(x,t) < 0$ and $f(x,t) > 1$, respectively, may introduce a substantial bias. In particular, an incorrect integration of $f(x,t)$ at the front, where $f(x,t)$ is small, might lead to an large error in the estimated velocity. However, when $f(x,t)$ is small so that $\gamma(t) \simeq \sqrt{\frac{2}{N} f(t)}$, this problem can be circumvented by integrating the noise term exactly [3], while integrating the rest of the equation deterministically [4].

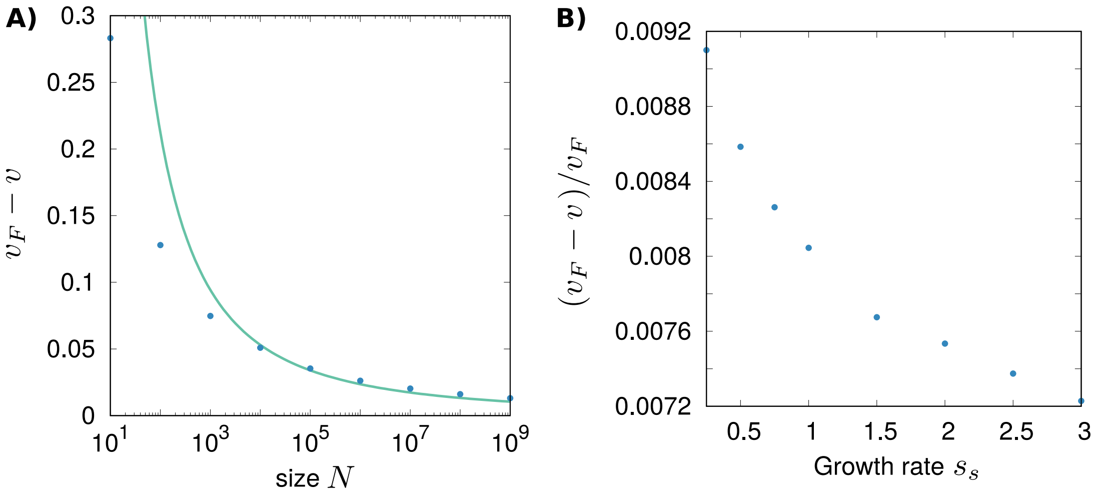

FIG. 1. **Size scaling and maximum error estimation for our integration method.** Panel A) shows the curve $4.5\,(\log N)^{-2}$ and the difference $v_F - v$ for $k = 0$, $\alpha = 0$, $s_s = 1$, and different system sizes. Panel B) shows $(v_F - v)/v_F$ for $N = 10^9$ and different growth rates employed in this work. These results suggest that our integration method is precise and have a maximum error of 0.0018%.

We follow this strategy and integrate the equation mixing two different algorithms, depending on the local value of $f(x,t)$:

- If $f(x,t) > \theta$: we employ the Milstein method (order 1). Defining $\beta(t) \equiv D\nabla^2 f(t) + \sigma(t) f(t)(1-f(t))$, the local field is updated according to the rule

$$f(x,t+dt) = f(x,t) + \beta(t)dt + \gamma(t)\Delta + \frac{1}{2}\gamma(t)\frac{\partial \gamma}{\partial f(t)}(\Delta^2 - dt) \tag{4}$$

being $\Delta = \sqrt{dt}g(t)$, where $g(t)$ is a Gaussian random variable with zero mean and unit variance, drawn independently at each time step.

- If $f(x,t) < \theta$ we employ a split-step numerical integration:

  1. *Non-linear and diffusion terms.* The term $\dot{f}(x,t) = D\nabla^2 f - \sigma(t)f^2$ is integrated by means of the Runge-Kutta method obtaining a first solution f^*.
  2. *Linear and stochastic terms.* The term $\sigma(t)f + \sqrt{\frac{2}{N}f}\xi(x,t)$ is integrated in an exact way, see [3]:

$$f(x,t) = r_{\text{Gamma}}\{r_{\text{Poisson}}\{\lambda f^*(x,t)e^{\sigma(t)t}\}\}/\lambda. \tag{5}$$

being $\lambda = 2\sigma(t)/[\gamma^2 e^{\sigma(t)t}]$, and r_{Gamma}, r_{Poisson} random variables from Gamma and Poisson probability distributions respectively, i.e. $Prob[r_{\text{Gamma}}(a) = z] = z^{a-1}e^{-z}/\Gamma[a]$ and $Prob[r_{\text{Poisson}}(a) = z] = a^z e^{-a}/z!$.

To check the precision of our method we integrated the stochastic equation (3) for $k = 0$, $\alpha = 0$, and different growth rates s_s and compared the results to the analytical Fisher velocity $v_F = 2\sqrt{Ds_s}$. For large population size N, the velocity v of the wave asymptotically goes as $v_F - v \simeq C\ln^{-2}(N)$ [5]. Our numerical integration is consistent with this asymptotic relation from $N \simeq 10^4$ (figure 1A) with a root-mean-square deviation of 0.002. We have also obtained the values $(v_F - v)/v_F$ for the different growth rates employed in this work to obtain an estimation of the maximum error (see figure 1B). Note that v_F is not the actual velocity of the finite system, so the relative error $(v_F - v)/v_F$ is, in fact, smaller. The maximum error is around 0.9%, that, considering the results of figure 1A) leads to an overestimated error of about 0.0018%.

[1] Richard Courant, Kurt Friedrichs, and Hans Lewy, "On the partial difference equations of mathematical physics," IBM journal of Research and Development **11**, 215–234 (1967).

[2] Kirill S Korolev, Mikkel Avlund, Oskar Hallatschek, and David R Nelson, "Genetic demixing and evolution in linear stepping stone models," Reviews of modern physics **82**, 1691 (2010).

[3] Ivan Dornic, Hugues Chaté, and Miguel A Munoz, "Integration of langevin equations with multiplicative noise and the viability of field theories for absorbing phase transitions," Physical review letters **94**, 100601 (2005).

[4] Haim Weissmann, Nadav M Shnerb, and David A Kessler, "Simulation of spatial systems with demographic noise," Physical Review E **98**, 022131 (2018).

[5] Éric Brunet and Bernard Derrida, "Effect of microscopic noise on front propagation," Journal of Statistical Physics **103**, 269–282 (2001).
