# Supplementary material for: Bet-hedging strategies in expanding populations
Source: PLoS Comput Biol. 2019 Apr 18;15(4):e1006529. doi: 10.1371/journal.pcbi.1006529 (PMC6490941; doi:10.1371/journal.pcbi.1006529)
Supplement: S2 Appendix — (PDF) [file pcbi.1006529.s002.pdf]

Supporting information: Bet-hedging strategies in expanding populations

Paula Villa Martín
Biological Complexity Unit, Okinawa Institute of Science and Technology Graduate University, Onna, Okinawa 904-0495, Japan

Miguel A. Muñoz
Departamento de Electromagnetismo y Física de la Materia and Instituto Carlos I de Física Teórica y Computacional, Facultad de Ciencias, Universidad de Granada, 18071 Granada, Spain

Simone Pigolotti*
Biological Complexity Unit, Okinawa Institute of Science and Technology Graduate University, Onna, Okinawa 904-0495, Japan.

(Dated: April 2, 2019)

EFFECT OF FINITE POPULATION SIZE FOR SPATIALLY VARYING ENVIRONMENTS

As in the temporally varying case (Section III.E), we study the effect of demographic stochasticity induced by the finite size of the population for spatially varying environments. In this case, the population front is described by the equation

$$\dot{f}(x,t) = D\nabla^2 f + \sigma(x) f(1-f) + \sqrt{\frac{2}{N} f(1-f)}\,\xi(x,t), \tag{1}$$

analogous to Eq. 10 of Section III.E. We numerically integrated this equation following the procedure described in S1. In this case, stochasticity slightly alters the deterministic prediction (see Fig. 1). Specifically, the asymptotic mean velocities v_M decay slightly faster with the fraction of risky individuals α. This implies that parameters such that the deterministic optimal strategy is $\alpha^* = 1$ can lead to a bet-hedging strategy with $\alpha^* < 1$ in the stochastic case. The lower limit for stochastic systems must still be equal to the deterministic case, $\tilde{s}_b > 2 - \tilde{s}_a$.

Despite this slight difference between the deterministic and stochastic systems, the main predictions described for spatially varying environments in the main text are still maintained.

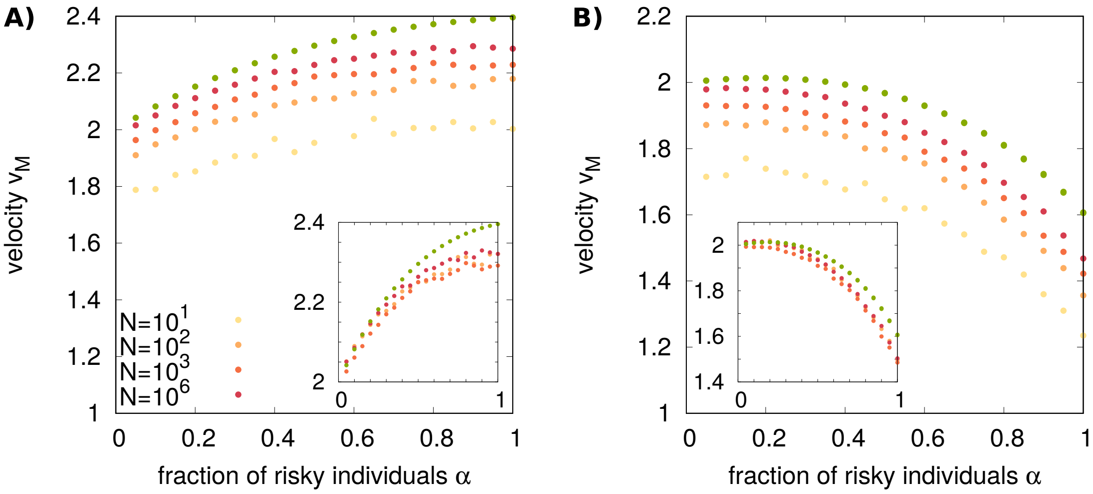

* simone.pigolotti@oist.jp

FIG. 1. **Optimal strategy with fluctuations induced by finite population size in spatially varying environments.** (A) Asymptotic mean velocities obtained by numerical integration of the stochastic Fisher (1) for $\tilde{s}_a = 0.75$, $s_s = 0.01$, $\tilde{s}_b = 3$ (yellow dot of Fig. 4) and different population sizes, shown in the figure legend. (B) Same for $\tilde{s}_a = 0.25$, $s_s = 1$, $\tilde{s}_b = 2$ (blue dot of Fig. 4). In both panels, the temporal switching rate of the environment is $k = 0.001$. Green dots corresponds to the results of the deterministic approach (Eq. 5) for $k = 0.001$. Insets show a collapse of the curves according to Eq. 11, with a fitted value of $C = 3$.
